# Exploring a Little-Known Pathway: Dermal Exposure to Phthalates in Indoor Air

**DOI:** 10.1289/ehp.123-A267

**Published:** 2015-10-01

**Authors:** Lindsey Konkel

**Affiliations:** Lindsey Konkel is a New Jersey–based journalist who reports on science, health, and the environment.

Certain phthalate esters used widely in vinyl plastics and other consumer products have been associated with impaired neurodevelopment,^1^ altered genital development,^2^ and respiratory problems^3^ in people. Studies of dermal absorption of phthalates have largely focused on direct contact of the skin with the chemicals, but some models predict that transdermal uptake directly from ambient air may be a potentially important route of exposure.^4^ In this issue of *EHP*, researchers confirm experimentally in humans that dermal uptake from indoor air may be a meaningful exposure pathway for some phthalates.^5^

In a series of experiments, six participants were exposed to elevated air concentrations of diethyl phthalate (DEP) and di(*n*-butyl) phthalate (DnBP). DEP is used as a solvent and carrier in personal care products such as cosmetics, perfumes, and shampoos.^6^ DnBP is used as a plasticizer in products including nail polish and adhesives.^7^

**Figure:**
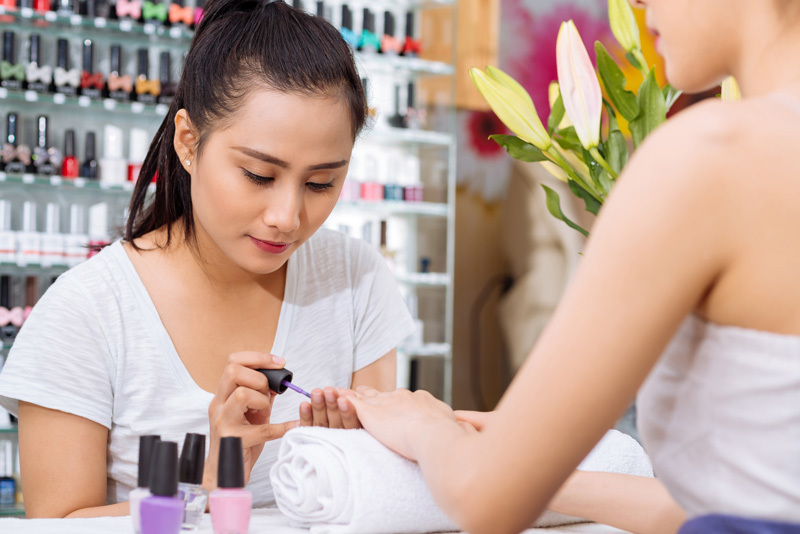
Previous studies have investigated uptake of phthalates via diet, dust ingestion, inhalation, and direct skin contact with phthalate-containing products. A new study suggests phthalates in ambient air may also pass through our skin. © Dragon Images/Shutterstock

“We chose these particular phthalates because they are ubiquitous in the indoor environment, and their metabolites are commonly found at high levels in human urine,” says lead study author Charles Weschler, an adjunct professor at Rutgers University.

The participants, all males between the ages of 27 and 66, each spent six hours on two different days in an exposure chamber. Phthalates were introduced into chamber air via aluminum plates coated with a phthalate-spiked paint. From 12 hours before the start of each experiment until 48 hours after exposure ended, each participant followed a strict diet and avoided using personal care products to minimize other exposures to phthalates—an approach that preliminary experiments proved to be successful.

On one of their exposure days, participants wore a hood and breathed filtered compressed air, which meant phthalate exposure was exclusively dermal. On the other day, they breathed chamber air and did not wear a hood, which resulted in both dermal and inhalation exposures. The researchers measured phthalate metabolites in urine samples collected over the 48 hours following each exposure period. To estimate uptake from inhalation only, they subtracted the estimated dermal uptake (based on hood days) from the total uptake estimated for nonhood days.

For both DEP and DnBP, both the dermal and inhalation pathways resulted in similar exposures.^5^ The participants stayed in the exposure chamber for just 6 hours, although Weschler’s previous dermal exposure models^8^ predict that the concentration of chemicals in the skin would continue to increase for about 36–48 hours. Weschler expects that dermal exposure would have been much higher relative to inhalation had the participants stayed in the chamber longer.

Only recently have scientists started to model dermal absorption of indoor air pollutants. “This study, as proof of concept, successfully confirms predictions about that pathway,” says Gerald Kasting, a professor of pharmaceutics and cosmetic science at the University of Cincinnati. Kasting was not involved in the study.

DEP and DnBP are not the only indoor organic pollutants predicted to have meaningful uptake via dermal absorption directly from the air. More than 30 semivolatile organic compounds commonly found indoors are predicted to have dermal uptakes similar to or greater than inhalation intake.^9^

“Our findings suggest that risk assessment models should not ignore the dermal pathway. There are other chemicals abundant in indoor air with the right physical properties … to move from air through skin to blood,” Weschler says.

In the current experiments, the participants wore only shorts so that the rest of their skin was exposed directly to the chamber air. One question raised by the research, Kasting says, is how clothing may impact transdermal chemical uptake. Weschler’s team, in a concurrent study,^10^ showed that a participant who donned fresh cotton clothing before entering the chamber had lower urinary phthalate levels than his bare-skinned counterparts. However, the same participant’s urine levels were higher than those of bare-skinned individuals when he put on clothing that had been stored in the chamber for days. “Clothing appears to be able to act either as a barrier or as an amplified source of exposure,” Weschler says.
